# An Infrared Near‐Sensor Reservoir Computing System Based on Large‐Dynamic‐Space Memristor with Tens of Thousands of States for Dynamic Gesture Perception

**DOI:** 10.1002/advs.202307359

**Published:** 2023-12-25

**Authors:** Jiejun Wang, Xinqiang Pan, Zebin Zhao, Yiduo Xie, Wenbo Luo, Qin Xie, Huizhong Zeng, Yao Shuai, Zeqian Song, Chuangui Wu, Wanli Zhang

**Affiliations:** ^1^ School of Integrated Circuit Science and Engineering University of Electronic Science and Technology of China Chengdu 611731 China; ^2^ National Key Laboratory of Electronic Thin Film and Integrated Devices University of Electronic Science and Technology of China Chengdu 611731 China

**Keywords:** analog memristor, dynamic gesture perception, Mott variable‐range hopping, near‐sensor computing, reservoir computing

## Abstract

To efficiently process the massive amount of sensor data, it is demanding to develop a new paradigm. Inspired by neurobiological systems, an infrared near‐senor reservoir computing (RC) system, consisting of infrared sensors and memristors based on single‐crystalline LiTaO_3_ and LiNbO_3_ (LN) thin film respectively, is demonstrated. The analog memristor is used as a reservoir in the RC system to process sensor signals with spatiotemporal characteristics. LN crystal structure stacked with oxygen octahedra provides favorable conditions for reliable Mott variable‐range hopping conduction, which provides the memristor with tens of thousands of reservoir states within a large dynamic range. With the characteristics, the analog sensor signals with high data fidelity can be directly fed to the memristive reservoir, and the spatiotemporal features can be separated and mapped. The system demonstrated a dynamic gesture perception task, achieving an accuracy of 99.6%, which highlights the great application potential of the memristor in signal sensor processing and will advance the application of artificial intelligence in sensor systems. Crystal ion slicing techniques are used to fabricate a single‐crystalline thin film for both the memristor and sensor, which opens up the possibility of realizing monolithic integration of a memristor‐based near‐sensor computing system.

## Graphical abstract

A novel infrared near‐sensor reservoir computing (RC) system constructed from an ion‐slicing LiTaO_3_‐based infrared array and ion‐slicing LiNbO_3_‐based memristor array is demonstrated. Thanks to the excellent capacities of the memristor reservoir, the infrared near‐senor RC system successfully and robustly implemented a dynamic gesture perception task with spatiotemporal feature fusion.

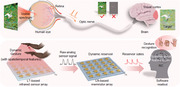

## 1 Introduction

**Figure 1:**
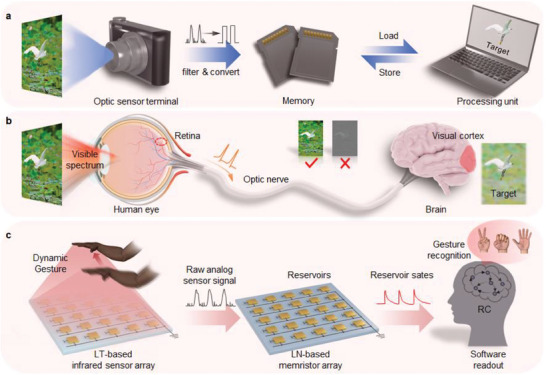
a) Schematic of traditional image processing architecture, realized by discrete optical sensor terminal with auxiliary modules (such as signal filters and ADCs), memory, and processing unit. Raw analog sensor signals are first filtered and converted to digital signals that are stored in memory. Processing units load data from memory and then transmit outputs back to memory for storage. b) Schematic of the human visual nervous system comprising the retina, optical nerve, and visual cortex in the human brain. It can process image signals with the feature of massively parallel in‐memory computation. However, the human visual system can only see objects in the visible spectrum and cannot see objects in non‐visible spectra, such as in the dark. c) Proposed infrared near‐senor RC system based on LT‐based infrared sensor array and LN‐based memristor array. The raw analog sensor signals of dynamic gestures collected by LT‐based infrared sensors are directly input to the LN reservoirs, and then product abundant reservoir states for gesture recognition realized by a software readout.

With the development of smart terminals and the Internet of Things, ubiquitous sensors in our daily life, are dramatically growing in both their number and rate of generating sensor data.^[^ ^1^, ^2^ ^]^ In conventional sensor signal processing systems, such as data‐intensive image processing tasks (**Figure** **1**a), the large amounts of image data detected by optical sensor terminals, including redundant background information or noises, must be filtered and then converted to digital data via analog‐digital‐converters (ADCs) and stored in memory, then transferred to processing units or cloud‐based computing systems for adapting the Von Neumann computing architecture.^[^ ^3^, ^4^ ^]^ Due to the physical separation of memory and computing unit (i.e., Von Neumann bottleneck),^[^ ^5^ ^]^ high energy consumption and high latency are inevitable. In addition, sensor signals detected from the real world usually have temporal features. However, it is difficult for some feedforward neural networks, such as convolutional neural networks (CNNs), to handle temporal tasks.^[^ ^6^ ^]^ These limitations hinder the further development of energy‐efficient and low‐latency sensor signal processing systems.^[^ ^7^, ^8^ ^]^

In human sensory systems, the nervous system composed of trillions of neurons and synapses can process sensory information efficiently.^[^ ^9^ ^]^ For example, in the human visual system (Figure 1b), objects are imaged in the retina and send nerve impulses through the optic nerve to the cerebral cortex, where dynamic visual information is processed in a highly parallel and energy‐efficient manner.^[^ ^10^ ^]^ Inspired by neurobiological systems, neuromorphic computing based on novel devices (including memristors) has emerged to break the Von Neumann bottleneck.^[^ ^11^ ^]^ Analog‐type memristors with abundant nonlinear dynamics can store information in the form of multilevel conductance states and process sensor signals in an analog fashion.^[^ ^12^, ^13^ ^]^ Hence, by combining the analog memristor with the sensor, bio‐inspired artificial sensory systems with energy‐efficient and low‐latency sensor signal processing ability can be constructed.^[^ ^14^, ^15^, ^16^ ^]^

As for the neural network, compared with some feedforward neural networks, recursive neural networks (RNNs) with cyclic connections offer an improved ability to process temporal data. More importantly, based on a specialized variant of RNN, memristor‐based reservoir computing (RC) systems recently have been proposed for processing various temporal tasks, such as spoken‐digit recognition^[^ ^17^ ^]^ and time‐series forecasting.^[^ ^18^, ^19^, ^20^ ^]^ Compared with the common RNNs, such an RC computational model, which only needs to train the readout layer of the network, can process the temporal data with a low computational cost and latency.^[^ ^21^, ^22^ ^]^ Therefore, it is promising to use a memristor as a physical reservoir to process sensor signals with low energy consumption and latency and to construct highly efficient artificial sensory systems.^[^ ^23^, ^24^ ^]^

However, as far as we know, the wavelength of reported RC‐based artificial vision systems was mainly concentrated on the visible spectrum^[^ ^25^ ^]^ and ultraviolet spectrum,^[^ ^19^, ^24^, ^26^ ^]^ and there are almost no reports on infrared near‐sensor computing systems, although infrared detection has shown irreplaceable advantages in the field of flame detection,^[^ ^27^ ^]^ thermal imaging^[^ ^28^ ^]^ and dynamic target recognition.^[^ ^29^ ^]^ The main reasons that hinder the development of infrared near‐sensor systems are the drawbacks of conventional infrared detectors, such as large volume and low compatibility with the fabrication process of memristor.^[^ ^30^, ^31^ ^]^ These limitations are adverse to the integration of a compact system and need extra efforts and costs to realize sensor signal tight coupling.^[^ ^32^ ^]^

In addition, for a memristive RC system, the reservoir (i.e., memristor) is not only expected to possess a nonlinear short‐term memory (STM) effect but also desired to have a large dynamic space with rich reservoir states (i.e., intermediate conductance states) to realize high‐dimensional mapping of input signal.^[^ ^17^, ^33^, ^34^ ^]^ Unfortunately, those existing emerging memristors are generally not satisfactory to meet the requirements of large dynamic space and rich reservoir states simultaneously.^[^ ^13^, ^35^ ^]^ Though some representative RC systems tried to solve this problem by taking advantage of device‐to‐device variances,^[^ ^18^ ^]^ additional mask process,^[^ ^17^ ^]^ or multiple reservoir layers,^[^ ^26^ ^]^ these approaches not only increase system complexity but also are not easily migrated to other artificial neural networks.^[^ ^6^, ^36^ ^]^ Moreover, the separation property is also required to separate originally distinct inputs into different classes and be insensitive to inessential signals, such as noises.^[^ ^34^ ^]^ It should be emphasized that the excellent separation property of the memristive reservoir is indispensable for processing sensor signals, because it ensures that the RC system can update weights according to target signals efficiently, and without interference from inessential signals such as noises (as shown in Figure S1, Supporting Information). Meanwhile, due to intrinsic disorders and uncontrollable internal dynamics in the memristive layer, traditional memristors based on polycrystalline/amorphous oxide thin film suffered well‐marked cyclic variations, device‐to‐device variations, and fluctuations, which hinder the yield of sufficiently distinguishable intermediate states to achieving high‐efficient network and training separate sensor signal inputs.^[^ ^12^, ^13^ ^]^

In this work, we designed and proposed a novel infrared near‐senor RC system with pyroelectric infrared sensor array based on single‐crystalline LiTaO_3_ (LT) thin film and memristors based on single‐crystalline LiNbO_3_ (LN) thin film, demonstrating a dynamic gesture perception task (Figure 1c). The pyroelectric infrared sensors based on single‐crystalline LT thin film and LN‐based memristors based on single‐crystalline LN thin film were prepared by the same manufacturing process, i.e., the crystal ion slicing (CIS) technique, which makes the fabrication process of two devices become compatible. Through defect engineering design, the LN memristor which exhibits reliable analog resistive switching and tunable STM characteristics was used as a dynamic reservoir. Thanks to the stable oxygen octahedral crystal structure of LN single crystal, the electron hopping process was carried out stably under electric field stimulation, yielding 20 000 reservoir states within a large dynamic range of 1891.78. By statistical analysis, the abundant reservoir states with tiny fluctuations allow sufficient margin to separate raw analog sensor signals and can effectively map the spatiotemporal features of original data. As a result, we demonstrated a dynamic gesture perception task with spatiotemporal feature fusion based on the infrared near‐senor RC system.

## 2 Results and Discussion

### 2.1 Analog Resistive Switching Characteristics of Memristor

**Figure 2:**
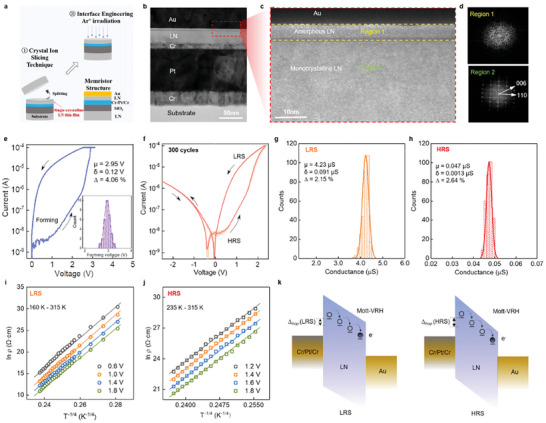
Analog resistive switching characteristics of LN memristor. a) Schematic of the key fabrication process of the memristor based on single‐crystalline LN thin film, including CIS and Ar^+^ beam irradiation. Inset: the cross‐section diagram of the device with a structure of Au/LN/Cr/Pt/Cr. b) Cross‐sectional TEM image of the LN memristor with a vertical Au/LN/Cr/Pt/Cr structure. Scale bar, 50 nm. c) High‐resolution TEM image of the region near the Au/irradiated LN interface (red dotted box in (b)) with the scale bar of 2 nm. d) The corresponding fast Fourier transform patterns of Region 1 and Region 2. e) Typical electroforming process of the memristor. Inset: the statistical analysis of the electroforming voltages of 30 different devices. f) *I–V* curves of 300 cycles. Statistical analysis on the LRS g) and HRS h) of the memristor over 300 cycles. The variation (∆) is the ratio of the standard deviation to the mean (δ/µ). Values of ln *ρ* as a function of *T* ^−1/4^ for different applied voltages under LRS i) and HRS j), respectively. k) Schematic diagrams of energy band alignment of LRS and HRS, respectively. The circles and light black balls represent trap sites and electrons, respectively. The arrows represent the electron hopping process.

**Figure** **2**a illustrates the schematic of the fabrication process of a memristor based on a single‐crystalline LN thin film (LN memristor). At the beginning, a multiple metal layer of Cr (10 nm)/Pt (100 nm)/Cr (30 nm) was sputtering on the He^+^‐implanted single‐crystalline LN wafer to act as the bottom electrode. Then the wafer was further bonded with another pristine LN wafer through SiO_2_ adhesion layer. After that, the single‐crystalline LN layer split from the single‐crystalline LN wafer, and a single‐crystalline LN thin film was obtained. After annealing and finer chemical mechanical polishing (CMP), the single‐crystalline LN thin film was further irradiated by Ar^+^ beam with an irradiated angle of 0° (*θ = 0°*) to reduce the thickness from 300 to 30 nm. Finally, the fabricated memristive device with a structure of Au/LN/Cr/Pt/Cr (see inset of Figure 2a) was formed after the Au top electrode with a diameter of 200 µm was fabricated by sputtering. For more details about the fabrication process see Figure S2 (Supporting Information) and Experimental Section.

Figure 2b displays the cross‐sectional transmission electron microscopy (TEM) image of the LN memristor. The memristor is configured in a vertical Au/LN/Cr/Pt/Cr structure, in which a LN thin film with a thickness of 30 nm is used as the resistive switching layer. In a high‐resolution TEM image, a uniform amorphous LN layer formed at the top electrode interface (Au/LN interface) by Ar^+^ beam irradiation can be observed, compared with the microstructure of pristine single‐crystalline LN thin film shown in Figure S3 (Supporting Information). The thickness of the amorphous layer is ≈5 nm (Region 1). Single‐crystalline LN was observed in Region 2, which indicates a good single‐crystalline feature of the residual LN layer. The results of the fast Fourier transform shown in Figure 2d further verify that the amorphous LN layer is formed on the single‐crystalline LN thin film layer after Ar^+^ irradiation. Based on systematic chemical composition analysis in our previous work, the previous results have already indicated that a large number of oxygen vacancy (*V*_o_) defects and suboxides of niobium elements were generated in the amorphous layer.^[^ ^37^, ^38^, ^39^ ^]^ It also has been proven that Ar^+^ irradiation is an effective defect engineering method to regulate the resistive switching behavior of memristors by modulating the *V_o_* s in the switching layer.^[^ ^40^, ^41^ ^]^

Figure 2e presents the typical electroforming process of the LN memristor. In addition, Figure 2e also displays the distribution of electroforming voltage of 30 different memristor devices. A tight voltage distribution (from 2.6 to 3.3 V) and low device‐to‐device variance (down to 4.06%) further confirm the device uniformity. After electroforming, the device shows extremely uniform analog resistive switching behavior over 300 cycles, which is illustrated in Figure 2f. The voltage sweeping sequence for the *I–V* measurements is 0 *V* → maximum of positive voltage (+*V*_max_) → 0 *V* → maximum of negative voltage (−*V*_max_) → 0 V. Figure 2g,h plot the statistical distributions of low resistance state (LRS) and high resistance state (HRS) of the device. The normal Gaussian distributions with ultra‐small standard deviations (δ), i.e., δ = 0.091 µS for LRS and δ = 0.0013 µS for HRS, respectively. The mean values (µ) of LRS and HRS are 4.23 and 0.047 µS, respectively. A quantitative analysis of the cycle‐to‐cycle variances (Δ *=* δ/µ) shows a value of 2.15% and 2.64% for LRS and HRS, verifying the excellently uniform resistive switching of the LN memristor.

In order to reveal the switching mechanism of the LN memristor, temperature‐dependent *I–V* curves at LRS and HRS were measured within a temperature range of 160–315 K (Figure S4, Supporting Information). By fitting with possible transport models in oxides, the *I–V* relationships are in good agreement with the trap‐controlled space‐charge‐limited current (SCLC) mechanism^[^ ^38^, ^42^, ^43^ ^]^ (Figure S5, Supporting Information). But in fact, trap‐controlled SCLC is a simplified physical mechanism, it is the contributor of Ohmic, Poole–Frenkel, and hopping conduction when these conductions reach certain magnitudes.^[^ ^44^ ^]^ Through further evaluation, the *I–V* relationship under HRS and LRS is well‐fitted with the Mott–VRH model as a linear dependence of ln *ρ* on *T* ^−1/4^, where *ρ* is the resistivity and *T* is the temperature (Figure 2i,j). The Mott‐VRH conduction is expressed as below:

(1) $$\rho \left(T\right)=\rho_{0}\;exp\;{\left(\frac{T_{0}}{T}\right)}^{1/4}$$

where *T*_0_ is the characteristic temperature, ρ_0_ is a resistivity parameter. Considering an amorphous layer rich in *V_o_* defects introduced by Ar^+^ beam irradiation, trap levels are distributed in the bandgap of LN crystal (≈4 eV). As shown in the energy band diagram, the trapped electrons undergo a hopping process under an electric field (Figure 1k). Compared with the hopping process under LRS (160–315 K), the trapped electrons under HRS need to acquire sufficient energy for the hopping transport at a higher temperature (up to 235 K), which might be due to field‐driven differential distributions of *V_o_* s.^[^ ^44^ ^]^ This phenomenon can be demonstrated by analyzing the density of states (DOS) at the Fermi level (*N*(*E*_F_)) and average hopping energy (Δhop). In the Mott–VRH regime, the *N*(*E*_F_) is related to the characteristic temperature *T*_0_ as^[^ ^45^, ^46^ ^]^

(2) $$N\left(E_{F}\right)=\frac{24}{\pi \;k_{B}\;T_{0}\;\zeta^{3}}$$

The Δhop is simply as^[^ ^45^, ^46^ ^]^

(3) $$\Delta \mathrm{hop}=\frac{1}{4}\;k_{B}T{\left(\frac{T_{0}}{T}\right)}^{1/4}$$

where  *k*_B_ is Boltzmann constant. ζ is localization length, which is a multiple of the minimum distance (*a*) between the hopping sites. Here, the minimum hopping sites can be attributed to *V*_o_s in LN, and *a* is ≈2.72 Å which corresponds to interatomic distances of O─O.^[^ ^47^, ^48^ ^]^ For our LN‐based device, the value of Δhop is evaluated at the representative temperature of 300 K. As a result, the DOS at Fermi level (*N (E_F_)* ≈8.32 × 10^16^ eV^−1^ cm^−3^) under LRS is higher over an order of magnitude than that of HRS (*N (E_F_)* ≈5.20 × 10^15^ eV^−1^ cm^−3^). Besides, the average hopping energy (Δ_hop_) with a value of 0.26 eV at 300 K guarantees electron hopping transport under LRS (_hop_ (LRS) = 0.26 eV), whereas higher hopping energy is required for HRS (Δ_hop_ (HRS) = 0.53 eV). Overall, the conduction mechanism in our LN memristor is dominated by Mott–VRH conduction, and the *V*_o_ defects in highly‐ordered oxygen octahedral of single‐crystalline LN thin film provide trap sites for electron hopping, which supports the reliable analog resistive switching characteristics of the device.

### 2.2 Dynamic Response Properties of LN Memristor for Reservoir Computing

**Figure 3:**
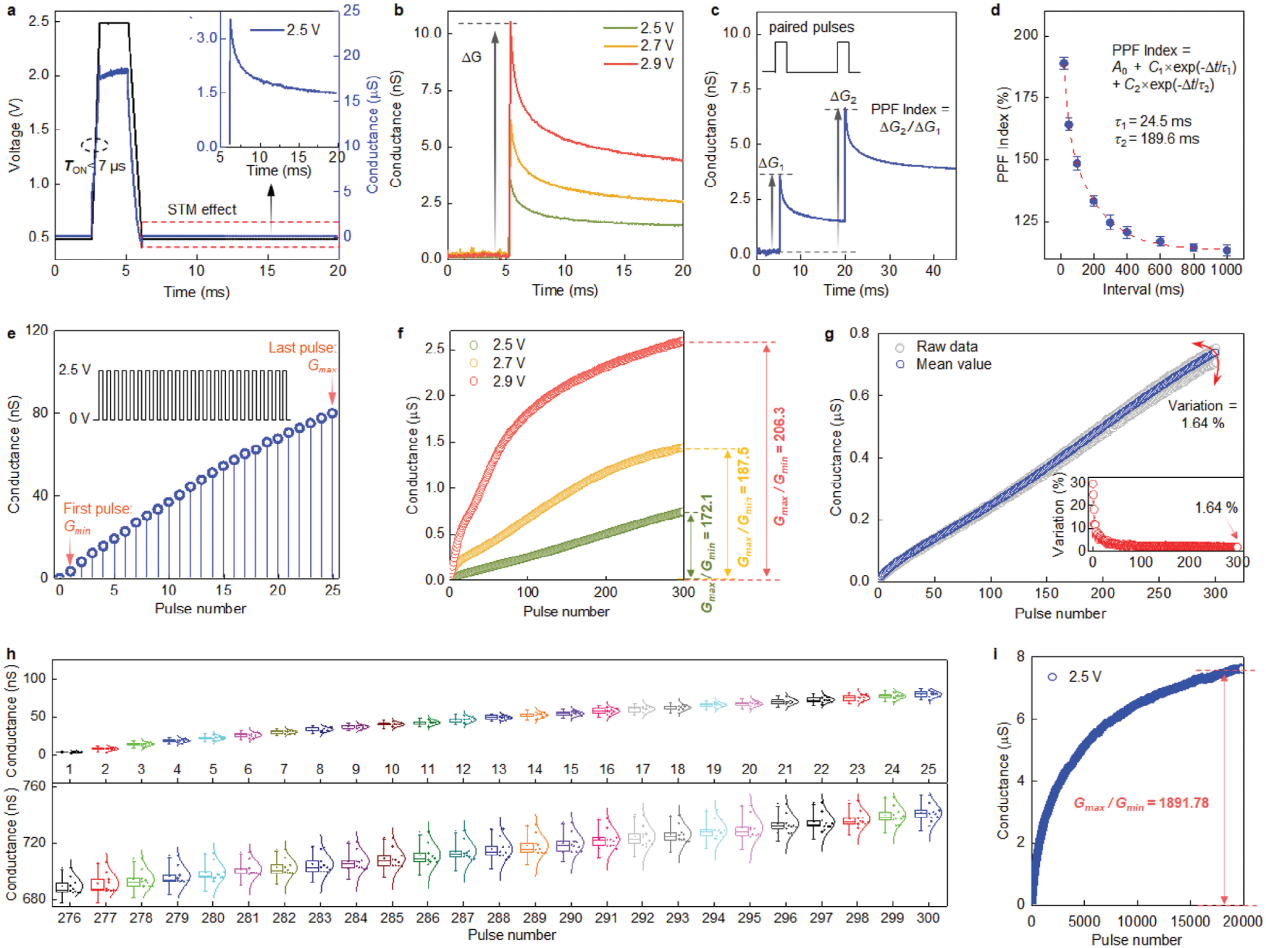
Dynamic responses of LN memristor for reservoir computing. a) Transient response of the LN memristor when stimulated by a voltage pulse (amplitude: 2.5 V, width: 2 ms). A read voltage pulse train (0.5 V, 50 µs) is used to study the device current before applying the stimulation and after removing the stimulation. The inset is a spontaneous decay process, which shows a nonlinear STM effect. b) Amplitude‐dependent dynamic response at the initial state and after being stimulated by a voltage pulse with different amplitudes (2.5/2.7/2.9 V). The response conductance (*ΔG*) after removing the stimulus is defined as the difference between maximum device conductance and initial value. c) PPF effect of the LN memristor. *ΔG*_1_ and *ΔG*_2_ denote the response conductance of the first stimulus and the second one in paired pulses, respectively. d) Dependence of PPF indexes on pulse interval (from 15 to 1000 ms), fitted by a double‐exponential function. Error bars: the standard deviation calculated from ten measurements. e) Dynamic reservoir states of the LN memristor are stimulated by a continuous voltage pulses sequence consisting of 25 voltage pulses with an amplitude of 2.5 V, width of 2 ms, and interval of 2 ms. The reservoir states are recorded by a read pulse after each write pulse is removed. Defining the conductance value measured after the first stimulus as *G*_min_ and the conductance value measured after the last pulse as *G*_max_. f) Amplitude‐dependent reservoir states changed with 300 successive pulse stimuli. The ratio of *G*_max_ to *G*_min_ (*G*_max_/*G*_min_) is adopted to estimate the dynamic range of reservoir. g) Cycling tests of the LN reservoir show excellent cycle‐to‐cycle repeatability during 300 successive pulse stimuli (variation: down to 1.64%). Inset is the variation of 300 reservoir states during ten cycles. The variation is calculated as the standard deviation‐to‐mean. h) The statistical results of the reservoir states in ten cycles for the initial 25 pulse stimuli and the final 25 pulse stimuli are depicted by a box error diagram and normal distribution. i) Tens of thousands of finely spaced reservoir states with a large dynamic range of 1891.78 obtained by a voltage pulse sequence consisting of 20 000 successive pulses (2.5 V, 2 ms).

In an RC system, the reservoir properties of the memristor directly determine the mapping quality of temporal inputs and significantly affect the system performance. **Figure** **3**a plots the transient response of the memristor when stimulated by a voltage pulse with an amplitude of 2.5 V (width is 2 ms). After ≈7 µs, the device conductance reaches a peak and then enters a spontaneous decay process as pulse removal. The conductance changes nonlinearly, and the spontaneous decay shows the STM effect. To further test the nonlinear STM characteristics of the LN reservoir, the amplitude‐dependent dynamic responses of the memristor at the initial state and after being stimulated by a voltage pulse were measured, as shown in Figure 3b. Obviously, the response conductance (*ΔG*) after the removal of the pulse stimulus is defined as the difference between maximum device conductance and initial value, which reveals an enhanced STM effect as the pulse amplitude increases. Applying voltage pulses of different duration times can also achieve tunably nonlinear STM properties (Figure S6, Supporting Information). Besides, a negative voltage pulse strategy is used for quickly resetting the conductance to the initial state to ensure the device's repeatable operation (Figure S7, Supporting Information). The nonlinear dynamics of the memristor can be further revealed by consecutive pulse stimuli. Typical short‐term plasticity of biological synapses, namely paired‐pulse facilitation (PPF), demonstrates the ability to process continuous temporal information. Hence, paired pulses were applied to the device and its dynamic response is presented in Figure 3c. During operation, the pulse is set to 2.5 V, and the pulse width is 2 ms. The interval of paired pulses is 15 ms. Taking *ΔG*_1_ as the response conductance of the first stimulus and *ΔG*_2_ of the second one, the corresponding *ΔG*_2_ is obviously higher than *ΔG*_1_, indicating the facilitation effect of synaptic plasticity. In terms of a ratio of *ΔG*_2_ to *ΔG*_1_, i.e., the PPF index, it is calculated as 188.6% in this case. This indicates that the LN memristor has the capability to realize nonlinear mapping of input signals.

To demonstrate the dependence of PPF on pulse interval, we then tested and plotted the PPF index under various intervals (Figure 3d). The results show that the PPF index decreases exponentially with increasing pulse intervals. The PPF index versus pulse interval can be fitted by a double‐exponential function as below:

(4) $$\mathrm{PPF}\;\mathrm{index}=A_{0}+C_{1}\times exp\left(-\frac{\Delta t}{\tau_{1}}\right)+C_{2}\times exp\left(-\frac{\Delta t}{\tau_{2}}\right)$$

where *A*_0_ is a preparameter, *C*_1_ and *C*_2_ are the facilitation factors and *τ*_1_ and *τ*_2_ with fitted values of 24.5 and 189.6 ms are the characteristic time constants related to relaxation times, respectively. As a result, a shorter pulse interval induces a higher PPF index or facilitation effect, making the STM generated by different inputs more distinguishable. Therefore, a continuous sequence consisting of 25 pulses with an interval of 2 ms was utilized to evaluate the potentiation process of the reservoir states (Figure 3e). Each reservoir state is recorded with a read voltage pulse with an amplitude of 0.5 V after each stimulus is removed. Results show that the reservoir state increases almost linearly with the increase in the number of pulse stimuli. Defining the conductance value of the first stimulus as *G*_min_ and the conductance value of the last pulse as *G*_max_, the ratio of *G*_max_ to *G*_min_ (*G*_max_/*G*_min_) can be adopted to estimate the dynamic range of the reservoir. Figure 3f presents the amplitude‐dependent reservoir states changed with 300 successive pulse stimuli. The results reveal that the dynamic range exceeds two orders of magnitude and increases further with the pulse amplitude. It is worth noting that the adjustable reservoir states with tiny fluctuations are beneficial to distinguish inputs for efficient mapping.

To further assess the impact of fluctuation and the uniformity of repeated operation, a cycling test consisting of 300 consecutive pulses was performed over ten cycles (Figure 3g; Figure S8, Supporting Information). After each cycle, the device is reset to its initial state (≈0.43 nA) by using a negative pulse strategy. Results show that the LN reservoir has excellent cycle‐to‐cycle repeatability, which is characterized by consistent linearity, close dynamic range (Figure S8, Supporting Information), and ultra‐low cyclic variation of reservoir states (down to 1.64%). A more detailed statistical analysis of reservoir states is shown in Figure 3h (1–25th pulses, 276–300th pulses). The small deviation and normal distribution of every state indicate the LN reservoir is capable of separating distinct inputs into different classes. Interestingly, the deviation of state distribution increases gradually with the increase in the number of pulse stimuli, which may be due to the accumulation effect of noise arising from intrinsic dynamics. In order to further demonstrate the capacity of LN reservoir, a voltage pulse sequence containing 20 000 successive pulses (2.5 V, 2 ms) is applied to the LN reservoir (Figure 3i). The obtained tens of thousands of reservoir states with an extremely large dynamic range of 1891.78 provide rich space to reflect the spatiotemporal feature information on different inputs. Meanwhile, 20 000 finely spaced reservoir states allow sufficient margins to separate each other (Figure S9, Supporting Information). In terms of energy consumption, it can be estimated by per pulse operation of the LN reservoir and calculated to be 12 picojoules (12 pJ = 2.5 V × 2.4 nA × 2 ms), indicating that the reservoir possesses energy‐efficient characteristics. Overall, the developed LN memristor reservoir exhibits excellent properties, including abundant nonlinear dynamics, STM effect, extremely large dynamic space, and tens of thousands of reservoir states, which guarantees the development of high‐performance RC systems for executing temporal‐dependent recognition tasks.

### 2.3 An Infrared Near‐Sensor RC System for Dynamic Gesture Perception

**Figure 4:**
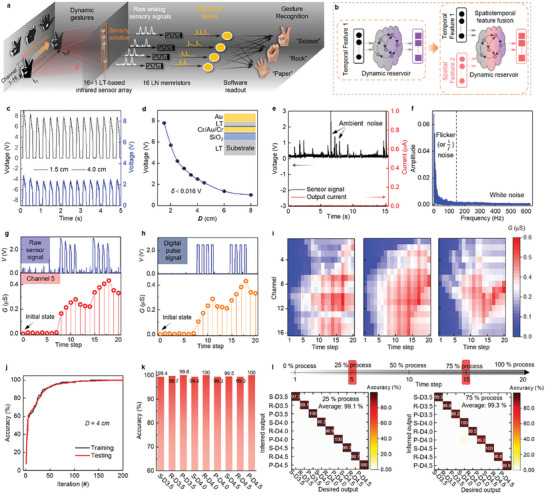
Memristor‐based infrared near‐sensor RC system for dynamic gesture perception. a) A conceptual schematic of the memristor‐based infrared near‐sensor RC system. The system consists of a 16 × 1 infrared sensor array based on single‐crystalline LT thin film, 16 memristors, and the software readout layer. The gesture moves parallel to the axis of sensing window at the exact speed (0.75 cm ^−1^ s) and varying detection distances (*D*). The moving process is split into 20 time steps. The sensor signals of three dynamic gestures (“Scissor,” “Rock,” and “Paper”) are recorded by an infrared sensor array mounted in a testing box with a sensing window. These raw analog sensor signals are then directly input into the LN memristor reservoir. Finally, the reservoir states collected by a customized FPGA controller are fed to the software readout layer for the recognition task. During performed dynamic gesture perception task, the system is completely in the dark environment to achieve infrared signal detection. b) Schematic of RC systems in which dynamic reservoir maps the inputs only with a single temporal feature (left column) or with spatiotemporal feature fusion (right column). c) Real‐time responses of the infrared sensor measured under a detection distance of 1.5 and 4 cm (*D* = 1.5 cm, *D* = 4 cm). d) Distance‐dependent peak values of the sensor signals under different detection distances (*D* = 1.5/2/2.5/3/3.5/4/4.5/6/8 cm). The standard deviation (*δ*) is calculated from 16 LT‐based infrared sensors. Inset is the cross‐section diagram of the infrared sensor with a structure of Au/LT/Cr/Au/Cr. e) A noise test of the infrared near‐sensor RC system in a real working environment. f) Noise spectrum of the raw sensor signals in (e) was analyzed through the Fourier transform. g) Output reservoir states obtained from memristors in Channel five after moving the “Scissor” gesture with a detection distance of 4 cm (*D* = 4 cm). The top column is the raw sensor signals collected by an infrared sensor. h) Output reservoir states are stimulated by corresponding digital voltage pulses as a reference. The top column is a digital voltage pulse sequence where voltage pulses are generated by Keithley 4200A‐SCS with a pulse measure unit (PMU). i) The 320 reservoir states (16 channels × 20‐time steps) of three dynamic gestures (“Scissor,” “Rock,” “Paper”). j) Evolution of recognition accuracy of the three dynamic gestures with a detection distance of 4 cm (*D* = 4 cm). k) Recognition accuracy of the three dynamic gestures moving at different detection distances (*D* = 3.5/4/4.5 cm). The abbreviation symbols (S‐D3.5, R‐D3.5, P‐D3.5, S‐D4.0, R‐D4.0, P‐D4.0, S‐D4.5, R‐D4.5, P‐D4.5) denote the nine possible recognition results, respectively. Taking “S‐D3.5″ as an example, it represents “Scissor” gesture moving at a detection distance of 3.5 cm (*D* = 3.5 cm). l) Schematic diagram of the different portions of raw sensor signals in the whole dynamic gesture process (Top column). The gesture moves through 20 time steps which correspond to the whole dynamic gesture process (100%). Confusion matrices of the predicted accuracy using 25% and 75% of the entire dynamic gesture process with different detection distances (*D* = 3.5/4/4.5 cm) (Bottom column).

To validate the ability of nonlinear mapping of real‐world sensor signals on LN reservoir, an infrared near‐sensor RC system based on LT pyroelectric infrared sensors and LN reservoirs was designed and demonstrated for a dynamic gesture perception task. The LT single crystal is a kind of multifunctional material with a similar structure to LN single crystal.^[^ ^49^ ^]^ It also belongs to a deformed perovskite structure stacked with oxygen octahedra and is one of the most promising materials for infrared detection due to its extremely high pyroelectric coefficient (up to 230 *µ*C cm^−2^ K).^[^ ^50^ ^]^ The pyroelectric output current (***i***_**p**_) can be expressed as $i_{p}=pA_{s}\frac{\Delta T_{p}}{dt}$,^[^ ^51^ ^]^ where ***p*** is the pyroelectric coefficient, ***A***_**s**_ is the sensing area, and $\frac{\Delta T_{p}}{dt}$ represents temperature alteration ratio. It can be seen that the ***i***_**p**_ strongly depends on the ***p*** and the $\frac{\Delta T_{p}}{dt}$. Thus, developing high‐quality single‐crystalline LT thin film is the guarantee to achieve a high pyroelectric coefficient and improve the sensitivity of temperature change. Here, we prepared a single‐crystalline LT thin film for the infrared sensor by the CIS technique which is the same as that used to fabricate a single‐crystalline LN thin film for the memristor (Figure S10, Supporting Information, inset of **Figure** **4**d, and Experimental Section). In our previous work, LT‐based pyroelectric infrared sensors have been designed and developed which show excellent pyroelectric properties.^[^ ^52^, ^53^ ^]^ For this work, the output current signals of the LT‐based infrared sensors are converted into analog voltage signals through a trans‐impedance amplifier (TIA) as the input signals of the memristive reservoir.

As shown in Figure 4a, the infrared near‐sensor RC system consists of a 16 × 1 infrared sensor array based on single‐crystalline LT thin film, 16 memristors based on single‐crystalline LN thin film, and the software readout layer (a 16 × 9 single‐layer perceptron). The sensor array is directly connected to memristive reservoirs to achieve tight coupling of signals through 16 channels. When the hand performs a certain gesture, the raw sensor signals collected from the LT‐based infrared sensor array are fed into the LN reservoirs and mapped to a high‐dimensional system. Since the infrared signals of moving gestures are recorded by the LT‐based infrared sensors in real time the raw sensor signals in different situations are time series data. Hence, the reservoir states output from LN reservoirs possess temporal characteristics. In this demonstration, the sensor signals of three classes of moving gestures (“Scissor,” “Rock,” and “Paper”) are recorded at a sampling frequency of 3.33 Hz. The LT‐based sensors are mounted in a testing box with a sensing window (see Figure S11, Supporting Information and Experimental Section). Corresponding to the chopper sampling frequency (3.33 Hz), gestures move parallel to the axis of the sensing window at the exact speed (0.75 cm ^−1^s) and are completed in 20 time steps. The sampling frequency of 3.33 Hz means that the interval of the sensor pulse sequence is 300 ms. Under this sampling strategy, the LN reservoir has a facilitation effect, that is, short‐term memory characteristic (corresponding PPF index as shown in Figure 2d). After high‐dimensional mapping by the LN reservoir, the dynamic reservoir states are collected by a customized Field‐programmable gate array (FPGA) controller to serve as the input of the readout layer. Figure S12 (Supporting Information) illustrates the test board and operational flow.

More importantly, since the detection distance of pyroelectric infrared sensors affects the temperature alteration ratio, sensor signals will vary with the distance between gestures and the sensing window (*D*). Therefore, the analog signals of dynamic gestures in different situations recorded by LT‐based infrared sensors contain temporal and spatial characteristics. In other reported memristive RC systems, the memristive reservoirs mapped the inputs only with temporal features to complete recognition or prediction tasks (Figure 4b (left column)), such as handwriting recognition,^[^ ^33^ ^]^ speech recognition,^[^ ^17^ ^]^ and chaotic system prediction.^[^ ^18^ ^]^ For this task, using an LN reservoir physically implements the fusion processing of temporal features and spatial features of the analog sensor signals. The large dynamic range and rich reservoir states of LN reservoir provide sufficient space for the implementation of spatiotemporal feature fusion (Figure 4b (right column)).

First, the properties of LT‐based infrared sensors are evaluated. As plotted in Figure 4c, the real‐time responses of the sensor are measured under a detection distance of 1.5 and 4 cm (***D*** = 1.5 cm, ***D*** = 4 cm). The LT‐based infrared sensor can detect targets effectively and output continuous analog signals. It should be noted that the larger peak of the initial sensor signal is because the target induces a higher temperature alteration ratio ($\frac{\Delta T_{p}}{dt}$) when it just enters the detection field and then recovers after ≈2 pulses. In addition, the sensor exhibits fast response times of ≈15 and 14 ms when the sensing window opens up or turns off, respectively (Figure S13, Supporting Information). Figure 4d shows the distance‐dependent peaks of sensor signals (***D*** = 1.5/2/2.5/3/3.5/4/4.5/6/8 cm). Obviously, a short detection distance (smaller ***D***) leads to a higher output signal.

In an actual environment, the sensor will be disturbed by various noises, and it will also lead to signal fluctuation. When these raw sensor signals as inputs feed to reservoir, the insensitivity of reservoir to these unessential fluctuations or noises is an important index of separation property for classifying similar inputs into the same class.^[^ ^34^ ^]^ Hence, a noise test of the near‐sensor RC system in a real‐working environment was carried out. In order to further assess the robustness of the system, we added interference of human activity (including people walking around and slight impact of the test board) during the experiment. The synchronous recording of sensor signals and reservoir outputs is shown in Figure 4e. Despite experiencing significant ambient noises (numerous signal spikes), the LN reservoir consistently produces stable output. Through noise spectrum analysis, in addition to ambient noise, Flicker noise (or *1*/*f* noise) and white noise can also be detected by the sensitive infrared LT‐based sensor, whereas LN reservoir can remain insensitive to these noises and remain stable (Figure 4f). The trapped electrons in LN crystals require sufficient energy to jump over the trap barrier into the conduction band or from one trap site to another to achieve Mott–VRH conduction (*∆*_hop_ (LRS) = 0.26 eV, *∆*_hop_ (HRS) = 0.53 eV). Therefore, we believe that the noises detected by the infrared sensor are inadequate to excite trapped electrons to complete Mott‐VRH transport so that the LN reservoir is insensitive to noises. Such a robust near‐sensor RC system significantly improves energy efficiency by self‐masking invalid raw data (e.g., various noises) in the noisy analog domain, which is especially important capacity in data‐intensive applications.^[^ ^8^ ^]^

Based on above analysis, the dynamic gesture perception task is implemented on the infrared near‐sensor RC system. Each gesture with a specific detection distance (***D*** = 3.5/4/4.5 cm) moves 200 times to generate the dataset, and the dataset is then divided into two groups: 120 randomly selected samples for training and the remaining 80 samples for testing. The total dataset consists of 1800 groups, including nine situations of three gestures with three detection distances. Take the “scissors” gesture with a detection distance of 4 cm (***D*** = 4 cm) as an example, the reservoir response to sensor signal trains in Channel 5 and Channel 13 are shown in Figure 4g and Figure S14 (Supporting Information), respectively. Due to nonlinear STM dynamics, the LN reservoir has the ability to distinguish the sensor signal sequences with different spatiotemporal features. During high‐dimensional mapping, the reservoir separates the valuable input signals into different classes, while it is insensitive to inessential noises, demonstrating good separation capacity. More importantly, compared with digital input (Figure 4h; Figure S14, Supporting Information), the analog sensor signals with high data fidelity directly feed to the LN reservoir can reflect the spatiotemporal features of raw data at full steam, whereas the large dynamic range with abundant reservoir states allows sufficient margin to distinguish each other. For every gesture movement, the 320 reservoir states (16 channels × 20‐time steps) of each gesture are implemented by LN reservoirs (Figure 4i). Intriguingly, the spatiotemporal information of sensor signal inputs is synchronously stored in the state matrix at each time step due to the STM property of memristive reservoir. Hence, the entire motion trajectory of dynamic gestures can be captured and recorded by this infrared near‐sensor RC system.^[^ ^54^ ^]^

In order to evaluate the recognition performance of the system, a commonly simple learning algorithm, i.e., single‐layer perceptron (SLP), that can implement the readout functions, is used in our demonstration. Evidently, the recognition accuracy of dynamic gestures (***D*** = 4 cm) reaches 100% after a few iterations (Figure 4j, 181 iterations for training and 133 iterations for testing). For a more complex perception task, the dynamic gestures move at different detection distances, characterized by the fusion of spatiotemporal features, which can also be recognized by the system with an average accuracy of 99.6% (Figure 4k). In addition, as the reservoir possesses the capability of mapping spatiotemporal features of different inputs, the near‐sensor RC system can be used to predict dynamic gestures before they are completed.^[^ ^18^ ^]^ As illustrated in Figure 4i (top column), the gesture moves through 20 time steps which correspond to the whole sequence (100%). When 25% or 75% portion of the dynamic gesture is recorded by LT‐based infrared sensors, LN reservoirs map the partial inputs and then output corresponding reservoir states to implement forecasting (Figure S15, Supporting Information). Figure 4i displays the predicted accuracy of 25% and 75% portion for nine situations (three dynamic gestures with different detection distances), and the average recognition rates are both over 99.1%. These results indicate the infrared near‐sensor RC system also has a good recognition capability for the dynamic targets even only with partial spatiotemporal information. Significantly, our infrared near‐sensor RC system directly receives raw analog sensor signals and produces analog reservoir states that are stored in the reservoir without any signal filter, ADC, memory buffering, and other auxiliary modules, which could considerably reduce the power consumption and complexity of the overall system.^[^ ^34^, ^54^ ^]^ In addition, the system requires only a few training resources, including a few datasets and training times, to achieve dynamic gesture recognition or prediction with spatiotemporal feature fusion. It is further verified that such near‐sensor RC architecture possesses energy‐efficient characteristics and is highly attractive for emerging applications, especially for energy‐efficient edge systems.^[^ ^55^ ^]^

At last, we need to emphasize that CIS technique employed in this work is a potential thin film transfer technique. In this work, both the structure of infrared sensor and the structure of memristor are metal‐insulator‐metal structures, as illustrated in Figure S10 (Supporting Information) and Figure 2b. The fabrication processes of the memristor and infrared sensor are almost the same, as discussed in the Experimental Section and shown in Figure S2 (Supporting Information). Optimistically speaking, by monolithically integrating the infrared sensor with the analog LN memristor through a synchronously uniform CIS process, a compact and reconfigurable sensing‐memorizing‐computing microsystem can be achieved, which can significantly improve the integration density in the future.

## 3 Conclusion

In conclusion, a novel infrared near‐senor RC system composed of pyroelectric infrared sensors based on single‐crystalline LT thin film and memristors based on single‐crystalline LN thin film has been proposed and demonstrated in this work. In order to process infrared sensor signals with spatiotemporal characteristics efficiently, the analog LN memristor has been used as a physical reservoir to realize RC. Thanks to the stable oxygen octahedral crystal structure of LN single crystal, reliable Mott‐VRH transport dominates the conduction mechanism, which guarantees excellent properties for high‐dimensional mapping of the infrared sensor signal. The proposed reservoir based on LN memristor exhibits tunably nonlinear STM characteristics, an extremely large dynamic range (with the G_max_/G_min_ ratio of 1891.78), tens of thousands of finely spaced reservoir states (20 000 states), and good separation properties. As a result, the LN reservoir has sufficient space and margin for mapping and separating the raw analog sensor signals with multiple spatiotemporal features. Finally, a dynamic gesture perception task with spatiotemporal feature fusion was demonstrated based on the robust memristor‐based infrared near‐senor RC system. In this work, the memristor‐based computation of the sensor signal can remain in the analog domain, which can further reduce energy consumption and latency by eliminating the need for conversion to and from digital signal. The proof of concept highlights the great potential of memristor in the highly efficient signal sensor processing sensory system in the future, which will pave the new way for the application of AI in sensor systems with high sensor signal processing ability. The CIS technique for the fabrication of versatile single‐crystalline thin films points out a new direction to explore integrated multifunctional near‐sensor computing systems.

## 4 Experimental Section

### Fabrication of Ion‐Slicing LN‐Based Memristor

The high‐quality single‐crystalline LN thin films were developed by the CIS technique (Figure S2, Supporting Information). i) A Z‐cut single‐crystalline LN wafer with a thickness of 500 µm was used as substrate. ii) A buried damaged layer was generated in another single‐crystalline LN wafer by high‐energy He^+^ implantation with an energy of 300 keV. The fluence of He^+^ ions was 8 × 10^16^ cm^−2^. iii) After implantation, a Cr/Pt/Cr (10/100/30 nm) multi‐layer, as the bottom electrode, was deposited by sputtering on the implanted wafer. Then, SiO_2_ bonding/insulating layers (≈2 µm) were deposited on the implanted wafer and pristine single‐crystalline LN wafer as substrate by Plasma Enhanced Chemical Vapor Deposition. iv) The two single‐crystalline LN wafers were directly bonded together at room temperature. v) The exfoliated LN thin layer was split under thermal annealing treatment at 250 °C. vi) After that, a typical thickness (≈300 nm) of the single‐crystalline LN thin film was fabricated after high‐temperature thermal annealing and fine chemical mechanical polishing (CMP). vii) Then, the interface engineering was conducted on the as‐prepared single‐crystalline LN thin film via Ar^+^ beam irradiation. Here, the acceleration voltage was 80 eV. After irradiation for 20 min, the thickness of LN thin film was reduced from 300 to ≈30 nm. viii) Au circular electrodes (a thickness of 200 nm) with a diameter of 200 µm were deposited by sputtering. Therefore, the final configuration of the presented LN memristor with a structure of Au/LN/Cr/Pt/Cr was realized. Finally, the LN memristors were wire‐bonded on a printed circuit board (PCB).

### Fabrication of Ion‐Slicing LT‐Based Infrared Sensor

CIS technique was also used for preparing the infrared sensor based on single‐crystalline LT thin film. The fabrication process is almost the same as the counterpart of memristor. Here, a Z‐cut single‐crystalline LT wafer with a thickness of 500 µm was used as substrate. The energy of He^+^ implantation is 350 keV. The influence value of He^+^ ion was 8 × 10^16^ cm^−2^. In addition, the bottom electrode of the LT‐based infrared sensor is Cr/Au/Cr with a thickness of 10/100/30 nm. After splitting and fine CMP, the single‐crystalline LT thin film was prepared with a thickness of 900 nm. After this, Au electrodes with a thickness of 500 nm were fabricated on the LT thin film by sputtering. Finally, commercial photosensitive polymer ink was spin‐coated on the wafer.

### Electrical Measurement and Material Characterization

The measurements of current–voltage (*I–V*) curves were performed by Keithley 4200A‐SCS with a source measure unit (SMU). Analog switching characteristics of memristors were measured with Keithley 2636 SourceMeter with a customized LabView Program and in Keithley 4200A‐SCS with a pulse measure unit (PMU). In addition, the samples were mounted in a cryostat for temperature‐dependent measurements of *I–V* curves. The morphology and cross‐section structures of the developed devices were investigated by scanning electron microscopy (SEM, Gemini) and transmission electron microscopy (TEM, Jeol JEM‐2100). Electron microscopy specimens were prepared by a focused ion beam (FIB) system (Helios NanoLab 600).

### Infrared Near‐Sensor RC System for Dynamic Gesture Perception

The dynamic gesture perception task was conducted on an optical test platform, which consists of an oscilloscope (Agilent, DSO‐X‐2012A), a Fresnel lens, a mechanical chopper (Isotech, Signal recovery model 5113E), a testing box with sensing window, and a slide rail with a ruler. Corresponding to the chopper sampling frequency (3.33 Hz), the gesture moves parallel to the axis of sensing window at the exact speed (0.75 cm ^−1^ s) and is completed in 20 time steps. Moving gestures (“Scissor,” “Rock,” “Paper”) are concentrated and adjusted to parallel light by the Fresnel lens and then recorded at a sampling frequency of 3.33 Hz by an infrared sensor array mounted in a testing box. The distance between gestures and sensing window (*D*) can be regulated by the slide rail with a ruler. During performed dynamic gesture perception task, the system was completed in a dark environment to achieve infrared signal detection. It should be noted that in order to concentrate the whole gesture by Fresnel lens, the distance from the dynamic gesture to the chopper is fixed (1.2 m), so the distance was used from the sensing window to the chopper to represent *D* in this work. When executing the dynamic gesture perception task, the customized FPGA controller connected with the LN reservoirs was used for recording the reservoir states in real‐time. Finally, the obtained reservoir states fed to readout function and realize gesture recognition or prediction. Here, a commonly simple learning algorithm, i.e., single‐layer perceptron, was used to implement the readout functions. The gradient descent rule with mean square error loss function and the Sigmoid activation function were used for algorithm implementation.

## Conflict of Interest

The authors declare no conflict of interest.

## Supporting information

Supporting Information

## Data Availability

The data that support the findings of this study are available from the corresponding author upon reasonable request.
