# Supplementary material for: An Infrared Near‐Sensor Reservoir Computing System Based on Large‐Dynamic‐Space Memristor with Tens of Thousands of States for Dynamic Gesture Perception
Source: Adv Sci (Weinh). 2023 Dec 25;11(6):2307359. doi: 10.1002/advs.202307359 (PMC10853732; doi:10.1002/advs.202307359)
Supplement: Supplementary file 1 — Supporting Information [file ADVS-11-2307359-s001.pdf]

ADVANCED SCIENCE
Open Access

Supporting Information

for *Adv. Sci.*, DOI 10.1002/advs.202307359

An Infrared Near-Sensor Reservoir Computing System Based on Large-Dynamic-Space Memristor with Tens of Thousands of States for Dynamic Gesture Perception

Jiejun Wang, Xinqiang Pan, Zebin Zhao, Yiduo Xie, Wenbo Luo, Qin Xie, Huizhong Zeng, Yao Shuai, Zeqian Song, Chuangui Wu and Wanli Zhang**

Supporting Information

An Infrared Near-Sensor Reservoir Computing System Based on Large-Dynamic-Space Memristor with Tens of Thousands of States for Dynamic Gesture Perception

Jiejun Wang, Xinqiang Pan, Zebin Zhao, Yiduo Xie, Wenbo Luo, Huizhong Zeng, Yao Shuai, Zeqian Song, Qin Xie, Chuangui Wu, Wanli Zhang*

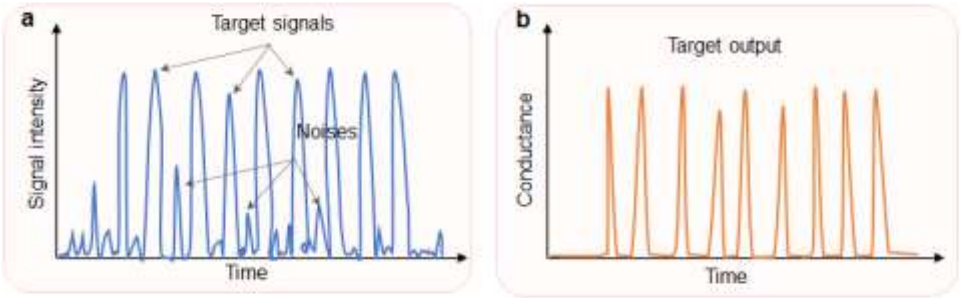

Figure S1. The separation property of memristor-based reservoir computing (RC) system. (a) Input signals. (b) Outputs of memristor-based RC system. Separation property refers to separating originally distinct inputs into different classes and being insensitive to inessential signals, such as noise. Separation property ensures that the RC system can update weights according to target signals efficiently, and without interference from inessential signals such as noises.

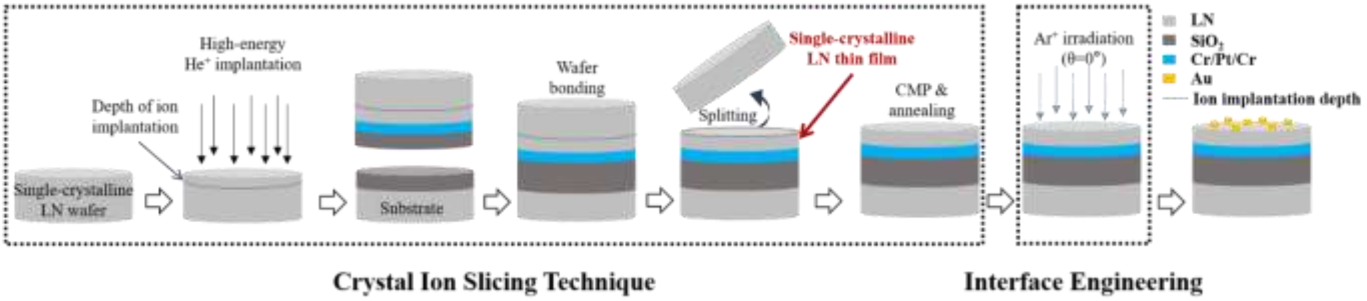

Figure S2. The fabrication process of the LN memristor. It includes the crystal ion slicing (CIS) technique and Ar^+ beam irradiation for interface engineering.

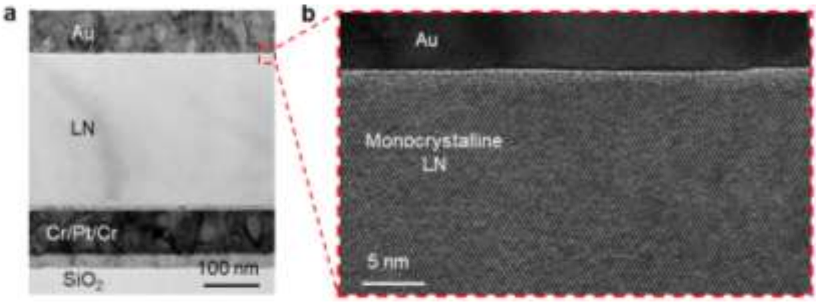

Figure S3. (a) The scanning transmission electron micrograph of the device based on pristine LN thin film. (b) High-resolution transmission electron microscopy image of the region near the Au/pristine LN interface.

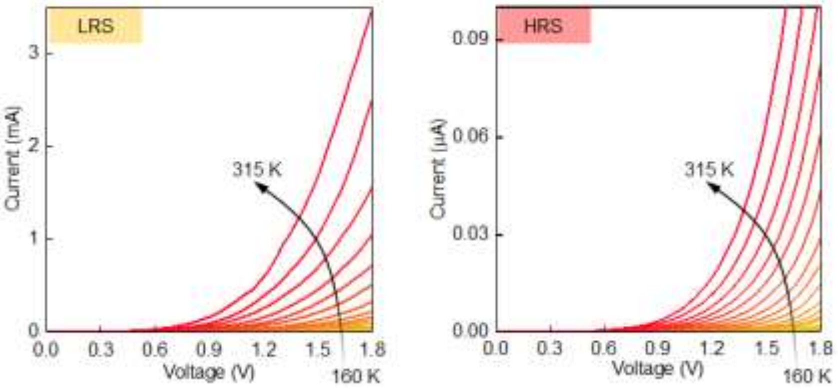

Figure S4. Temperature-dependent I-V sweeps of the LN memristor when the device is at LRS and HRS.

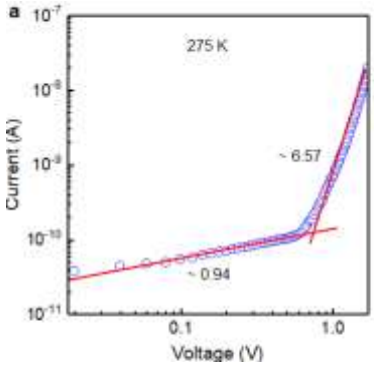

Figure S5. Double-logarithmic plot of the I-V curve when the LN memristor at HRS (at room temperature). The I-V relationships are in good agreement with trap-controlled space-charge-limited current (SCLC) mechanism. However, trap-controlled SCLC is not a single physical mechanism, it generally includes contributions from Ohmic, Poole-Frenkel, and hopping, and it is a condition or threshold for behavior when all of these contributors reach a certain magnitude [1]. Hence, the finely defined conduction mechanisms need to be carefully investigated.

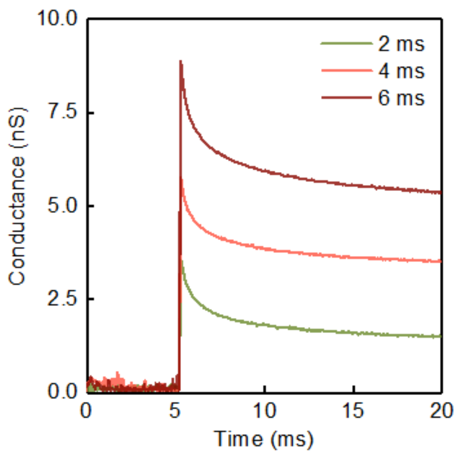

Figure S6. Duration-dependent dynamic responses after being stimulated by a voltage pulse with different duration times (2/4/6 ms). The pulse amplitude is fixed at 2.5 V.

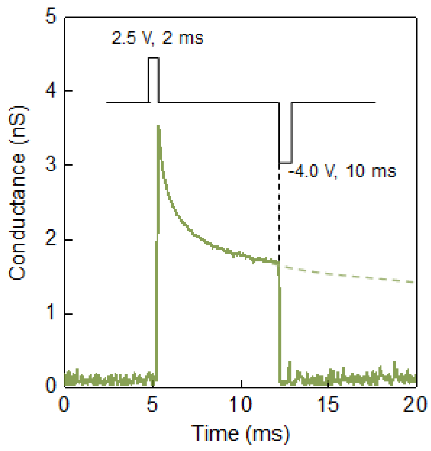

Figure S7. A negative voltage pulse strategy (-4.0 V, 10 ms) is used for quickly resetting the conductance to initial state for ensuring device's repeatable operation.

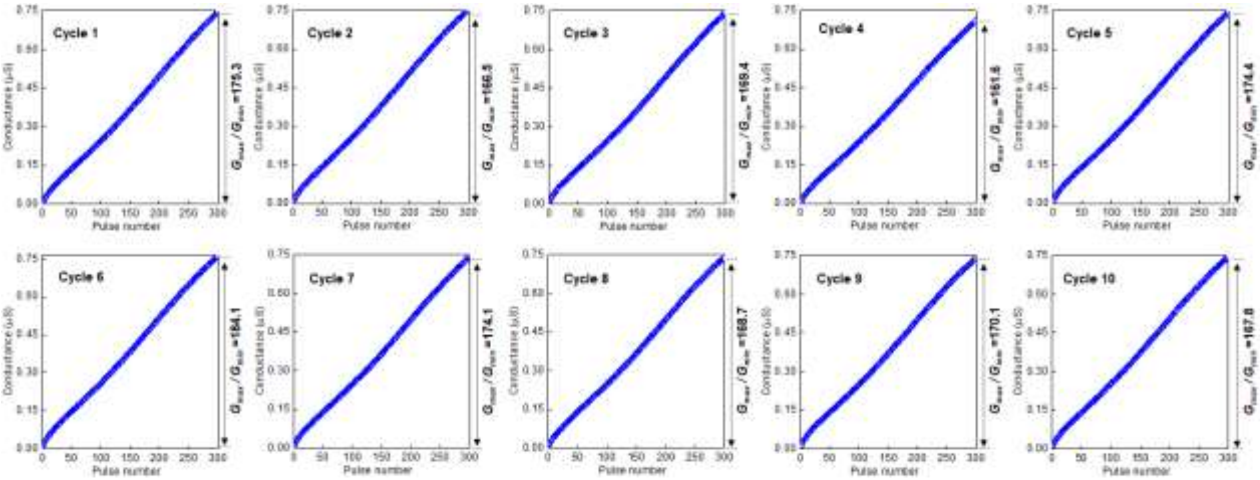

Figure S8.10 Cycle-to-cycle tests consisting of 300 consecutive pulses. The LN reservoir has excellent cycle-to-cycle repeatability, which is characterized by consistent linearity, close dynamic range, and ultra-low cyclic variation of reservoir states.

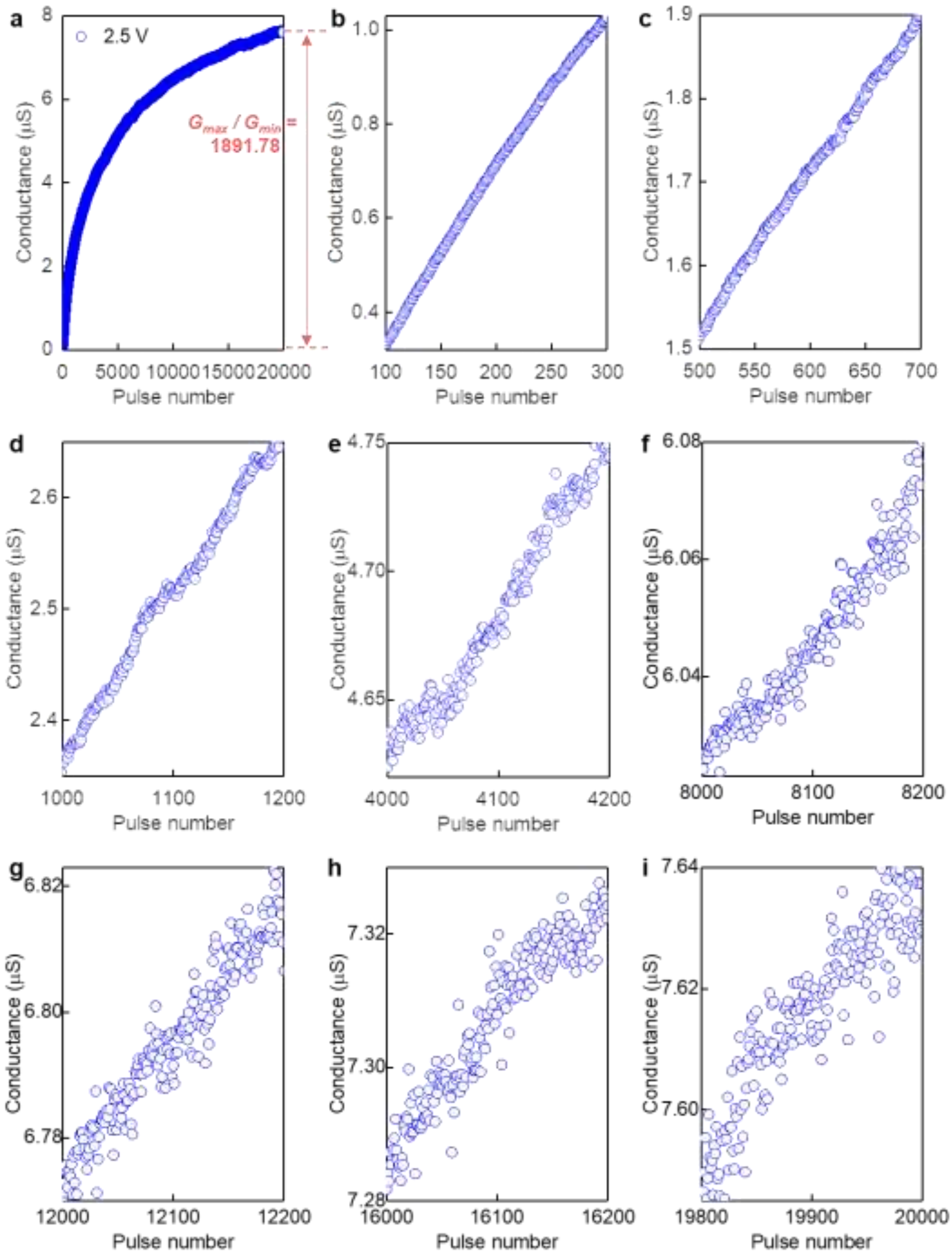

Figure S9. (a) The tens of thousands of finely spaced reservoir states obtained by a voltage pulse train containing 20000 successive pulses (2.5 V, 2 ms). (b)-(i) Enlarged plots of the reservoir states when the programmable pulses are in the range of 100-300th, 500-700th, 1000-1200th, 4000-4200th, 8000-8200th, 12000-12200th, 16000-16200th, and 19800-20000th. The results show that the LN reservoir has an extremely large dynamic range with tens of thousands of finely spaced reservoir states.

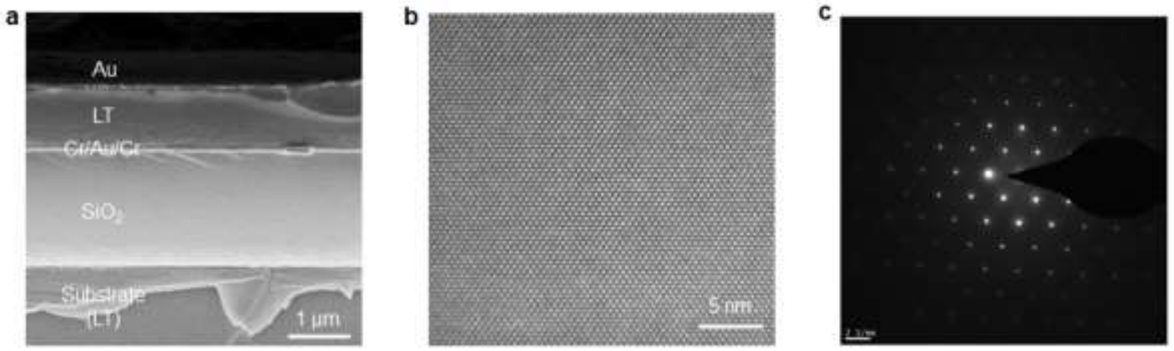

Figure S10. (a) Cross-sectional scanning electron microscope images of LT-based infrared sensor. The infrared sensor has a structure of Au/LT/Cr/Au/Cr based on a 900-nm LT thin film. Scale bar, 1 μm. (b) High-resolution transmission electron microscopy image of the single-crystalline LT thin film. Scale bar, 5 nm. (c) The corresponding electron diffraction pattern of the single-crystalline LT layer.

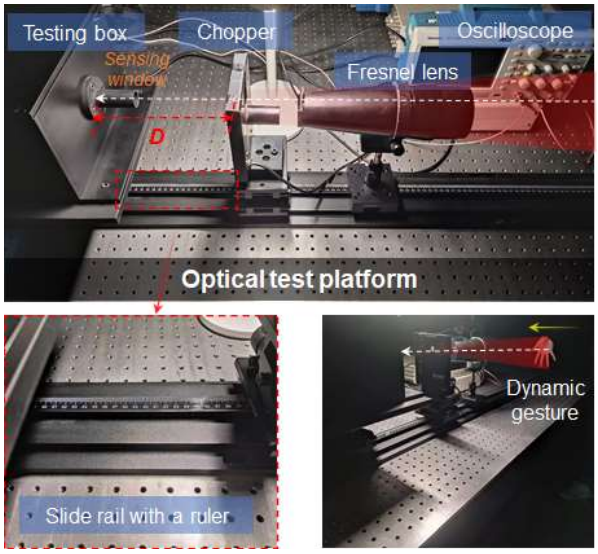

Figure S11. (a) Optical image of the test platform for infrared near-sensor RC system. The test platform includes an oscilloscope, a Fresnel lens, a chopper, a testing box with sensing window, and slide rail with a ruler. Corresponding to the chopper sampling frequency (3.33 Hz), the gesture moves parallel to the axis of sensing window at the exact speed (0.75 cm/s) and is completed in 20 time steps. Moving gestures (“Scissor”, “Rock”, “Paper”) are concentrated and adjusted to parallel light by the Fresnel lens and then recorded at a sampling frequency of 3.33 Hz by an infrared sensor array mounted in a testing box. The distance between gestures and sensing window (***D***) can be regulated by the slide rail with a ruler. During performed dynamic gesture perception task, the system is completely in the dark environment to achieve infrared signal detection. It should be noted that in order to concentrate the whole gesture by Fresnel lens, the distance from the dynamic gesture to the chopper is fixed (1.2 m), so we use the distance from the sensing window to the chopper to represent ***D*** in this work.

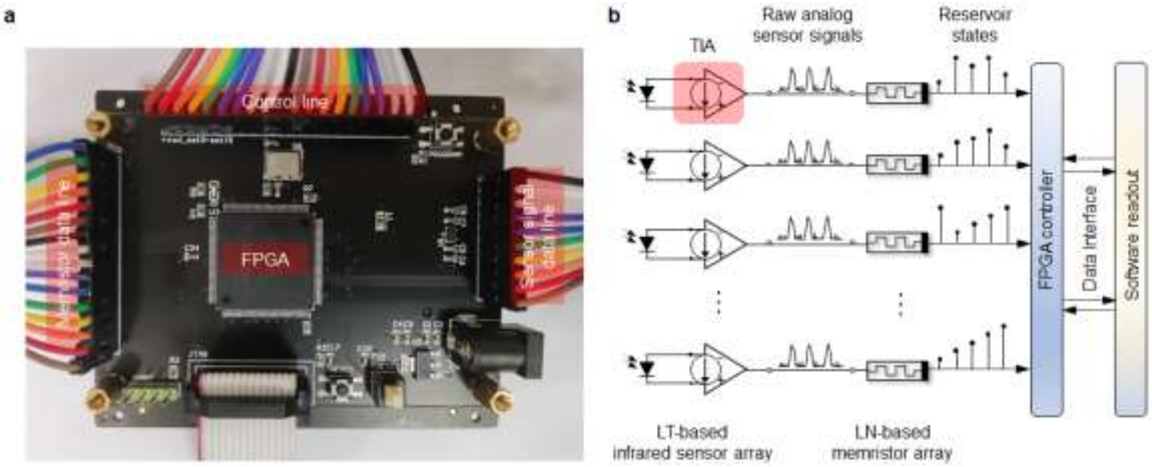

Figure S12. (a) Photograph of the test board. (b) Operational flowchart of the proposed infrared near-sensor RC system for dynamic gesture perception. It consists of an LT-based infrared sensor array with trans-impedance amplifiers (TIAs), an LN-based memristor array, and the software readout layer (PC). FPGA controller is used for reservoir state collection. The infrared near-sensor RC system can directly receive raw analog sensor signals and produce/store analog reservoir states without any signal filter, analog-digital converter (ADC), memory buffering, and other auxiliary modules.

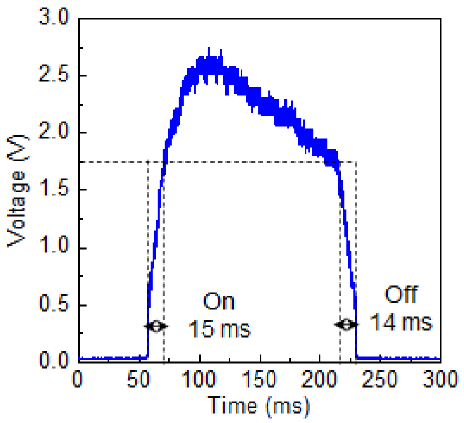

Figure S13. Response time of the LT-based infrared sensor. (a) The sensor shows an ultrafast response time around of 14.5 ms when sensing window opens up (On) and turns off (Off).

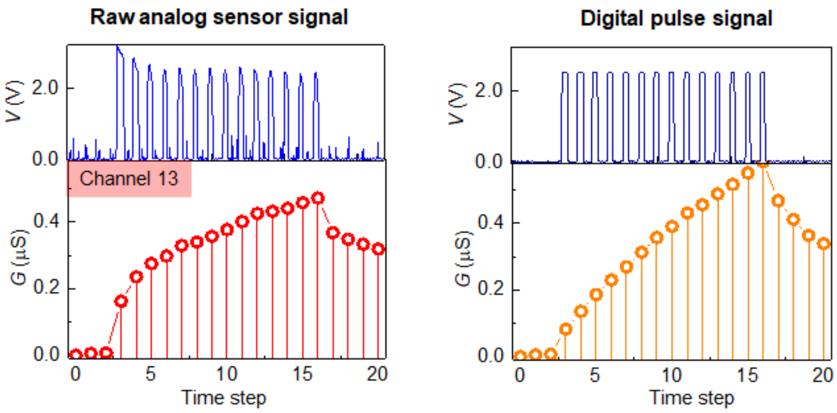

Figure S14. Left column: output reservoir states obtained from memristors in Channel 13 after moving the "Scissor" gesture with a detection distance of 4 cm (***D*** = 4 cm). The top column is the raw sensor signals collected by LT-based infrared sensor. For comparison, the output reservoir states stimulated by digital pulses are shown in the right column. The top column is a digital pulse sequence where voltage pulses are generated by Keithley 4200A-SCS with a pulse measure unit (PMU)). Compared with digital input, the raw analog sensor signals with high data fidelity directly fed to LN reservoir can reflect the spatiotemporal features of original data at full steam, whereas the large dynamic range with abundant reservoir states allows sufficient margin to distinguish each other.

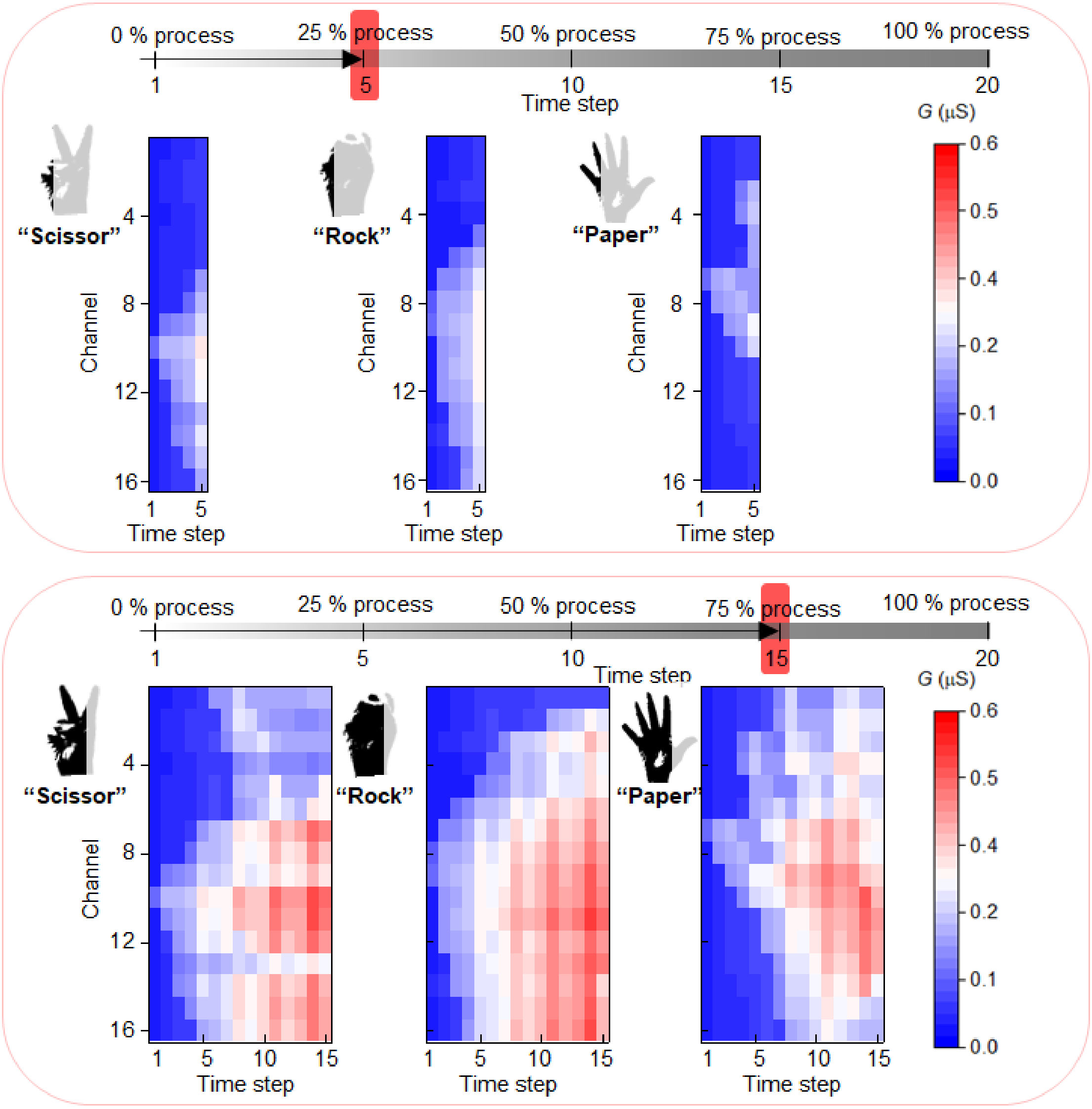

Figure S15. LN reservoirs map the partial inputs and then output corresponding reservoir states to implement forecasting when 25% and 75% of the three dynamic gestures are recorded by LT-based infrared sensors.

Reference

[1] H. J. H. Ma, J. F. Scott, *Phys Rev Lett* **2020**, 124, 146601.
